# Severe hypertriglyceridemia and hypercholesterolemia accelerating renal injury: a novel model of type 1 diabetic hamsters induced by short-term high-fat / high-cholesterol diet and low-dose streptozotocin

**DOI:** 10.1186/s12882-015-0041-5

**Published:** 2015-04-11

**Authors:** Liang He, Lili Hao, Xin Fu, Mingshu Huang, Rui Li

**Affiliations:** National Shanghai Center for New Drug Safety Evaluation and Research, 201203 Shanghai, China; College of Life Science and Technology, Southwest University for Nationalities, 610041 Chengdu, Sichuan China; State Key Laboratory Breeding Base for Zhejiang Sustainable Plant Pest Control, Agricultural Ministry Key Laboratory for Pesticide Residue Detection, 310021 Hangzhou, Zhejiang China; Institute of Quality and Standard for Agro-products, Zhejiang Academy of Agricultural Sciences, 310021 Hangzhou, Zhejiang China

**Keywords:** High-fat, High-cholesterol, Streptozotoncin, Hyperlipidemia, Hypertriglyceridemia, Hypercholesterolemia, Diabetes, Hamsters, Renal injury

## Abstract

**Background:**

Hyperlipidemia is thought to be a major risk factor for the progression of renal diseases in diabetes. Recent studies have shown that lipid profiles are commonly abnormal early on type 2 diabetes mellitus (T2DM) with diabetic nephropathy. However, the early effects of triglyceride and cholesterol abnormalities on renal injury in type 1 diabetes mellitus (T1DM) are not fully understood and require reliable animal models for exploration of the underlying mechanisms. Hamster models are important tools for studying lipid metabolism because of their similarity to humans in terms of lipid utilization and high susceptibility to dietary cholesterol and fat.

**Methods:**

Twenty-four male Golden Syrian hamsters (100–110 g) were rendered diabetes by intraperitoneal injections of streptozotocin (STZ) on consecutive 3 days at dose of 30 mg/kg, Ten days after STZ injections, hamsters with a plasma Glu concentration more than 12 mmol/L were selected as insulin deficient ones and divided into four groups (D-C, D-HF, D-HC, and D-HFHC), and fed with commercially available standard rodent chow, high-fat diet, high-cholesterol diet, high-fat and cholesterol diet respectively, for a period of four weeks.

**Results:**

After an induction phase, a stable model of renal injury was established with the aspects of early T1DM kidney disease, These aspects were severe hypertriglyceridemia, hypercholesterolemia, proteinuria with mesangial matrix accumulation, upgraded creatinine clearance, significant cholesterol and triglyceride deposition, and increasing glomerular surface area, thickness of basement membrane and mesangial expansion. The mRNA levels of sterol regulatory element binding protein-1c, transforming growth factors-β, plasminogen activator inhibitor-1, tumor necrosis factor-α and interleukin-6 in the D-HFHC group were significantly up-regulated compared with control groups.

**Conclusions:**

This study presents a novel, non-transgenic, non-surgical method for induction of renal injury in hamsters, which is an important complement to existing diabetic models for pathophysiological studies in early acute and chronic kidney disease, especially hyperlipidemia. These data suggest that both severe hypertriglyceridemia and hypercholesterolemia can accelerate renal injury in the early development of T1DM.

**Electronic supplementary material:**

The online version of this article (doi:10.1186/s12882-015-0041-5) contains supplementary material, which is available to authorized users.

## Background

Hyperlipidemia is a metabolic syndrome characterized by diverse lipid profiles (e.g. hypertriglyceridemia, hypercholesterolemia, and familial combined hyperlipidemia) [1], and is emerging as an independent risk factor for progression of renal disease in diabetes [2,3]. Findings from basic and clinical studies strongly suggest that hyperlipidemia can lead to glomerulosclerosis and tubulointerstitial fibrosis and induce renal injury by promoting the intrarenal generation of reactive oxygen species, glomerular infiltration of monocytes and macrophages, and podocyte damage [4]. However, these findings were obtained at stages with extensive renal injury and may have represented secondary effects.

Diabetes has been described as a major global health problem and rapidly growing cause of death and disability [5]. At least 50%, and up to as high as 90% of patients with type 1 diabetes mellitus (T1DM) and type 2 diabetes mellitus (T2DM) develop kidney disease, traditionally termed diabetic nephropathy, the disease burden being greater with T1DM [6-8]. Lipid profiles are commonly abnormal in the early stages of T2DM, observed as a temporal pattern that correlates with the presence of diabetic nephropathy [9]. Accumulating data from several large scale trials of patients with T2DM also point to early lipid abnormality as a major independent risk factor for the development of diabetic nephropathy [10], but the early effects of triglyceride and cholesterol abnormalities on renal injure in T1DM are not fully understood and the mechanisms underlying this need further exploration. One of the reasons is the lack of reliable animal models mimicking severe hypertriglyceridemia and hypercholesterolemia of human T1DM to assist in the understanding of the earlier incidence of diabetic nephropathy.

There have previously been some studies on investigation models of T1DM with exacerbation of diabetic kidney disease, such as renin overexpression, knockout of the bradykinin B2 receptor, and decorin deficiency [11-13]. The patterns of disease initiation and development in most of these studies do not appear to be analogous to the clinical situation in humans. Besides these transgenic animals, the widespread established techniques for induction of renal injury in mice are mostly dependent on surgical interventions, which lead to interstitial fibrosis by infiltration of macrophages and tubular cell death by apoptosis and necrosis, and 5/6 nephrectomy [14]. There are also several limitations, including a substantial mortality rate when not performed adequately, non-reversible, and phenotypic alterations related to the surgical procedure rather than impaired kidney function [15]. In attempts to circumvent these obstacles, several studies have reported that rodent models (mice and rats) fed a high fat or high cholesterol diet have been used to investigate the nephropathy associated with hyperlipidemia [16,17]. Mice and rats are not better than hamsters for reproducing hyperlipidemia. This is because hamsters have a unique and distinct hepatic lipid metabolism with a greater plasma lipid concentration than rats, and have cholesteryl ester transfer protein activities in plasma, which are very similar to that of humans [18,19]. Similarities between hamsters and humans are also obtained on the effects of dietary cholesterol on plasma lipid profiles [20].

Based on the available evidence, there are few studies that have specifically investigated the effects of severe hypertriglyceridemia and hypercholesterolemia on renal tissues in the early development of T1DM. The effects and mechanism of hyperlipidemia on renal injury require further investigation. Therefore, the objectives of the present study were to establish a novel, non-transgenic, and non-surgical animal model induced by a short-term high-fat/high-cholesterol diet and low-dose streptozotocin(STZ) treatment in Golden Syrian hamsters, and to explore the mechanisms underlying severe hypertriglyceridemia and hypercholesterolemia producing accelerated renal injury in this model.

## Methods

### Ethics statement

All animal procedures were approved by the Institutional Committee on the Use of Live Animals in Teaching and Research at Zhejiang Academy of Agricultural Sciences. ‘Principles of Laboratory Animal Care’ (NIH Publication No.85-23, revised 1996) were followed (Additional file 1).

### Chemicals and reagents

STZ, pioglitazone and glipizide were purchased from Sigma-Aldrich Chemical Co. (Skbio Life Sciences Technology, Beijing, China). Rat insulin ELISA kitS were purchased from Westang Biotechnology Co. (Shanghai, China). Glucose assay kits, cholesterol assay Kits, and triglyceride assay Kits were produced from BioSino Bio-technology and Science Co.(Beijing, China). Commercial fed a standard rodent chow (4% fat, 24% protein and 4.5% crude fiber), high-fat (15% fat) diet, high -cholesterol (0.5% cholesterol) diet, and high-fat and high-cholesterol (15% fat and 0.5% cholesterol) diet were purchased from Institute of Laboratory Animal Sciences , Chinese Academy of Medical Sciences. BCA protein assay kits were purchased from Bio-Rad Company (Bio-Rad Biotechnology, Beijing, China).

### Hamsters

Male Golden Syrian hamsters were purchased from Beijing Vital River Laboratory Animal Technology Co.,Ltd and bred at the Zhejiang Academy of Agricultural Sciences Laboratory Animal Centre. Hamsters aged 2 months old and weighing 100–110 g were used for experiments. All animals had free access to commercially available standard rodent chow and water. Animals were singly housed in plastic cages(370 × 215 × 170 mm) under controlled atmosphere (23 ± 1°C, 45 ± 5% relative humidity), fresh air exchange and 12/12 h light/dark cycle.

### Determination of threshold dose of STZ on hamsters producing insulin deficient

Twenty-four hamsters were rendered diabetes by intraperitoneal injections of STZ on 3 consecutive days, at dose of either 20 mg/kg, 30 mg/kg, 40 mg/kg or 50 mg/kg once daily in 0.05 M citrate buffer (pH 4.5). The control group was given 0.05 M citrate buffer (pH 4.5) without STZ. All groups (n = 6 in each group) were fed a standard rodent chow for 10 days after injection. Blood samples were collected to determine the effects of different doses of STZ on plasma glucose (Glu) and body weight.

### Model of hamsters induced by STZ

Ten days after intraperitoneal STZ injections, hamsters with a plasma Glu concentration more than 12 mmol/L were selected as insulin deficient ones for subsequent experiments [21]. Twenty-four STZ-treated hamsters were randomly divided into four groups (n = 6 in each group) according to the type of diet they recceived: either standard rodent chow (D-C), high-fat diet (D-HF), high-cholesterol diet (D-HC) or high-fat and high-cholesterol diet (D-HFHC). Twenty-four control hamsters were also divided into four groups (n = 6 in each group) according to the different diets they received(C-C, C-HF, C-HC or C-HFHC). All groups were fed for 4 weeks. Twenty-four hours urine samples were collected using metabolic cages before hamsters were anesthetized with pentobarbital sodium and exsanguinated by cutting the abdominal aorta.

### Determination of high-fat/high-cholesterol feeding and STZ-treated type1 diabetic hamsters through anti-diabetic compound effects

To determine the type of diabetes in high-fat/high-cholesterol fed and STZ-treated hamsters, classes of anti-diabetic drugs, insulin sensitizer (pioglitazone) and insulin secretagogue (glipizide), were used to treat on this model. Ten days after STZ administration was followed by 4 weeks of dietary manipulation, high-fat/high-cholesterol and STZ-treated hamsters were randomly divided in three groups of six hamsters per group (Glu >12 mmol/L). Diabetic hamsters were orally treated with pioglitazone (10 mg/kg once daily for 7 days) and a single dose of glipizide (5 mg/kg). The control group was orally given vehicle 1% Na-CMC (2 ml/kg). Blood samples were collected at 3 h after administration of the vehicle or test compounds to determine the effects of the drugs on plasma biochemical parameters: plasma Glu, total cholesterol (TC) and triglyceride (TG) levels in the treated animals.

### Measurement of plasma biochemical parameters

Blood samples were taken from the venous retroorbital plexus o hamsters after an overnight fast under pentobarbital sodium anesthesia (50 mg/kg). Plasma Glu, TC and TG levels were determined using enzymatic methods. Insulin was assayed with a rat insulin ELISA kit. Plasma and urinary creatinine concentrations were measured by an automated technique using the Jaffe method. Creatinine clearance is expressed in milliliters per minute.

### Measurement of urinary protein excretion

Twenty four hours urine samples were collected individually using metabolic cages. Urine was centrifuged at 3000 × g for 10 min, and the supernatant was collected. Urinary protein content was measured using protein assay kits. All samples were assayed in triplicate and mean values were used.

### Renal histological analysis

The left kidney was embedded in paraffin and cut into 3 μm thick sections for staining with Periodic acid-Schiff (PAS) and Masson’s trichrome. The kidney was also embedded in OCT compound (Tissue-Tek; Sakura Finetek USA) for cryostat sections, then Oil Red O staining was performed. Quantitative morphometric analysis of glomeruli was performed as described previously [22]. One section was used for measurement of glomerular area for each hamster. Each kidney of each animal was trimmed on the same place during the preparation of the slide. Fifty different glomeruli from the cortical area of each kidney were observed and images were taken using a digital microscope (BH-2; Olympus, Tokyo, Japan). For every investigated glomerulus, total glomerular area and glomerular tuft area were determined by tracing the outline of the Bowman’s capsule and the tuft, respectively, using Image-Pro Plus 4.5 soft ware (Media Cybernetics, Silver Spring, MD, USA). This analysis was conducted in a double blind manner.

### Lipid extraction, total RNA extraction and real-time PCR

At the end of the experiments, hamsters were killed and kidneys were harvested. The right kidney was removed and immediately placed in liquid nitrogen for total RNA extraction and renal lipid extraction. Total kidney lipid was extracted using the method of Bligh *et al.* [23]. TC and TG were measured as described above. Total RNA from the kidney was extracted using TRI Reagent (Molecular Research Center, USA) and first-strand cDNA was generated using a reverse transcription kit (Invitrogen, USA). In 25 μl reverse transcription reaction system, 5 μg of total RNA was used for each sample. Quantitative real-time PCR was performed in 35 cycles using an opticon continuous fluorescence detection system (MJ Research, Waltham, MA) with SYBR green fluorescence (Molecular Probes, Eugene, USA). All samples were quantitated using the comparative CT method for relative quantitation of gene expression, normalized to β-actin and 18S [24,25]. The primers used are listed in Table 1. The following primer pairs were designed using an online version of primer 3 software (http://bioinfo.ut.ee/primer3/) through analysis of available golden hamster genome sequences from NCBI. The quantitative real-time PCR was done in triplicates. The PCR conditions were as follows: incubate at 94°C for 5 minutes 5; 35 cycles of 94°C for 30 seconds, 55°C for 30 seconds, and 72°C for 30 seconds; incubate at 72°C for 7 minutes; perform melting curve from 65°C to 95°C: read every 0.2°C; hold for 3 sec between reads; incubate at 4°C for 30 min.

**Table 1:** **Primers for real-time PCR**

**Primer**	**Forward**	**Reverse**
TNF-α	CCTCCTGTCCGCCATCAA	CACTGAGTCGGTCACCTTTCT
SREBP-1c	GCACTTTTTGACACGTTTCTTC	CTGTACAGGCTCTCCTGTGG
PAI-1	CCTCACCAACATCTTGGATGCT	TGCAGTGCCTGTGCTACAGAGA
IL-6	TCGGAGGTTTGGTTACAT	GGAGGCATCCATCATTTA
TGF-β	CAAGGACCTCGGCTGGAAG	GCGCACGATCATGTTGGAC
VEGF	TCTTCAAGCCGTCCTGTG	TGCGGATCTTGGACAAAC
β-actin	TCAGAAGGACTCCTATAGTGG	TCTCTTTGATGTCACGCACG
18S	GGAAGGGCACCACCAGGAGT	TGCAGCCCCGGACATCTAAG

TNF-α: tumor necrosis factor-α; SREBP-1c: sterol regulatory element binding protein-1c; PAI-1: plasminogen activator inhibitor-1; IL-6: interleukin-6; TGF-β: transforming growth factors-β; VEGF: vascular endothelial growth factor.

### Statistics analysis

All results are expressed as means ± SD. Statistical comparison between groups were performed using 2-way ANOVA followed by a Fisher test. A P value ≤ 0.05 was considered statistically significant.

## Results

### Determination of threshold dose of STZ on hamsters producing insulin deficient

Intraperitoneal injection of STZ (30 mg/kg, 40 mg/kg and 50 mg/kg) on 3 consecutive days caused hyperglycemia (fasting blood glucose ≥ 12 mmol/L) in all hamsters. The 20 mg/kg dose of STZ did not produce significant hyperglycemia (Table 2). All STZ-treated hamsters exhibited a slight reduction in body weight, and some died within 10 days of STZ (40 mg/kg and 50 mg/kg) administration (data not shown). High-fat and/or high-cholesterol diet in combination with a low dose of STZ (30 mg/kg) was chosen for the generation of severe hypertriglyceridema and hypercholesterolemia in T1DM models for subsequent studies.

**Table 2:** **Plasma Glu levels after 10 days of STZ treatment**

**Group**	**N**	**Dose of STZ**	**B.W**	**Glu**
		**(mg/kg)**	**(g)**	**(mmol/l)**
STZ-20	6	20	110 ± 6^*^	8.6 ± 3.2
STZ-30	6	30	108 ± 5^*^	16.2 ± 2.5^**^
STZ-40	6	40	106 ± 4^*^	16.9 ± 3.3^***^
STZ-50	6	50	102 ± 6^**^	17.2 ± 4.9^***^
Control	6	0	116 ± 4	7.2 ± 2.5

^***^P<0.001, ^**^P<0.01, ^*^P<0.05 vs control group.

### Plasma biochemical analysis

**Figure 1:**
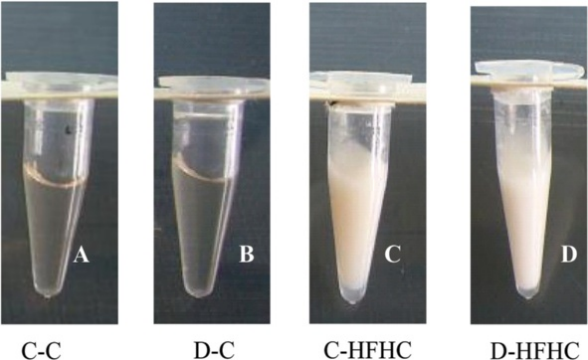
Appearance of plasma after 4 weeks STZ-treatment in combination with a chow diet or high-fat/high-cholesterol diet. C-C(**A**),D-C(**B**),C-HFHC(**C**) and D-HFHC(**D**).

In response to STZ-induced (30 mg/kg) diabetes, all groups of diabetic hamsters had a modest reduction in plasma insulin levels and exhibited hyperglycemia (fasting blood glucose ≥12 mmol/L). Body weights of the four diabetic groups (D-C, D-HF, D-HC, and D-HFHC) were significantly lower than those of the corresponding control groups (C-C, C-HF, C-HC, and C-HFHC). In non-diabetic hamsters fed a high-fat and/or high -cholesterol diet for 4 weeks, plasma TG and TC levels increased slightly (Table 3). In the diabetic groups, plasma TG and TC levels increased modestly in hamsters fed a high-fat or high-cholesterol diet. Hamsters in the D-HFHC group developed severe hypertriglyceridemia and hypercholesterolemia, the plasma sample of this group showed a milky appearance (Figure 1), in which plasma TG and TC levels reached 110 mmol/L and 75 mmol/L, respectively (Table 3).

**Table 3:** **Glu, TG and TC levels for each group 4 weeks after a high fat and /or high cholesterol diet**

**Group**	**N**	**B.W**	**Glu**	**TG**	**TC**	**Insulin**
		**(g)**	**(mmol/l)**	**(mmol/l)**	**(mmol/l)**	**(ng/ml)**
C-C^a^	6	111 ± 7	7.2 ± 1.3	1.4 ± 0.7	4.3 ± 0.6	1.58 ± 0.17
C-HF^a^	6	141 ± 4	8.0 ± 1.5	1.6 ± 0.6	3.8 ± 0.5	1.55 ± 0.12
C-HC^a^	6	115 ± 4	6.4 ± 0.6	1.6 ± 0.6	7.1 ± 0.6^**^	1.56 ± 0.05
C-HFHC^a^	6	139 ± 4	9.0 ± 0.9	7.8 ± 3.3^**^	26.6 ± 11.4^**^	1.59 ± 0.07
D-C^b^	6	98 ± 16^*^	20.1 ± 5.8^***^	1.8 ± 0.7	4.3 ± 1.1	1.12 ± 0.05^**^
D-HF^b^	6	97 ± 11^***^	21.7 ± 6.5^***^	6.1 ± 3.1^**##^	8.3 ± 4.5^*#^	1.18 ± 0.14^**^
D-HC^b^	6	94 ± 1^**^	19.6 ± 1.7^***^	3.8 ± 1.1^**##^	30.8 ± 9.5^**##^	1.18 ± 0.06^***^
D-HFHC^b^	6	100 ± 8^***^	16.3 ± 3.9^***^	116.7 ± 69.2^***###^	75.8 ± 49.5^***###^	1.17 ± 0.07^***^

^a^ non-diabetic hamsters; ^b^ diabetic hamsters.

^***^P<0.001, ^**^P<0.01, ^*^P<0.05 vs non-diabetic counterparts(C-C,C-HF,C-HC and C-HFHC).

^###^ P<0.001, ^##^ P<0.01, ^#^ P<0.05 vs D-C group.

### Determination of high-fat/high-cholesterol feeding and STZ-treated T1DM hamsters through anti-diabetic compound effects

**Figure 2:**
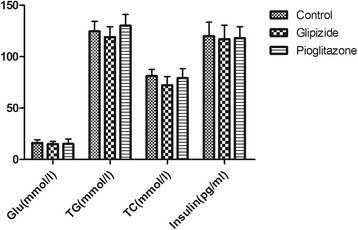
Effect of anti-diabetic compounds on various biochemical parameters in high fat/high cholesterol and STZ-treated hamsters. Values are mean ± SD (n = 6).

Both antihyperglycemic compounds (pioglitazone and glipizide) were not validated for the high-fat/high-cholesterol fed and STZ-treated (30 mg/kg, administered intraperitoneally) diabetic hamster model. Oral administration of pioglitazone for 7 days did not significantly reduce plasma Glu, TG, TC and insulin levels. When compared with vehicle-treated diabetic hamsters (Figure 2). Likewise, oral administration of glipizide (5 mg/kg) also failed to elicit any significant effect on these biochemical parameters (Figure 2).

### Kidney functions

**Figure 3:**
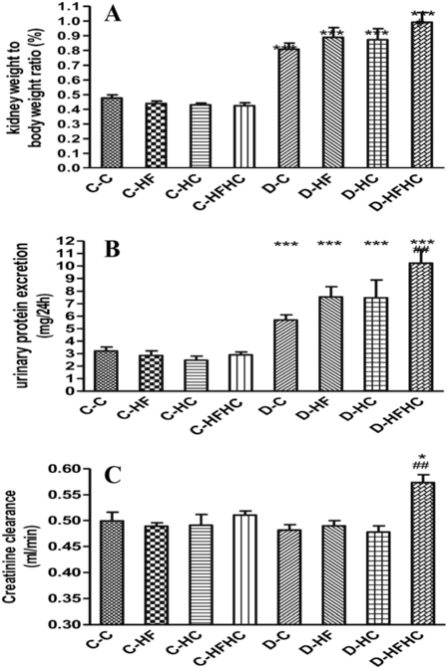
Kidney weight to body weight ratio **(A)**, urinary protein excretion **(B)**, and creatinine clearance **(C)** of hamsters from each group. n = 6 in each group. ^***^P< 0.001, ^**^P< 0.01, ^*^P< 0.05,diabetic vs non-diabetic counterparts; ^###^ P< 0.001, ^##^ P< 0.01, ^#^ P< 0.05 , diabetic with different diets vs D-C group.

All diabetic groups fed with a high-fat and/or high cholesterol diet had renal hypertrophy (Figure 3) and urorrhagia (data not shown) compared with non-diabetic groups. Urinary protein excretion increased significantly in the D-HFHC group compared with the other diabetic groups (P<0.01) (Figure 3). Creatinine clearance increased in the D-HFHC group only (Figure 3).

### Renal lipid deposition

**Figure 4:**
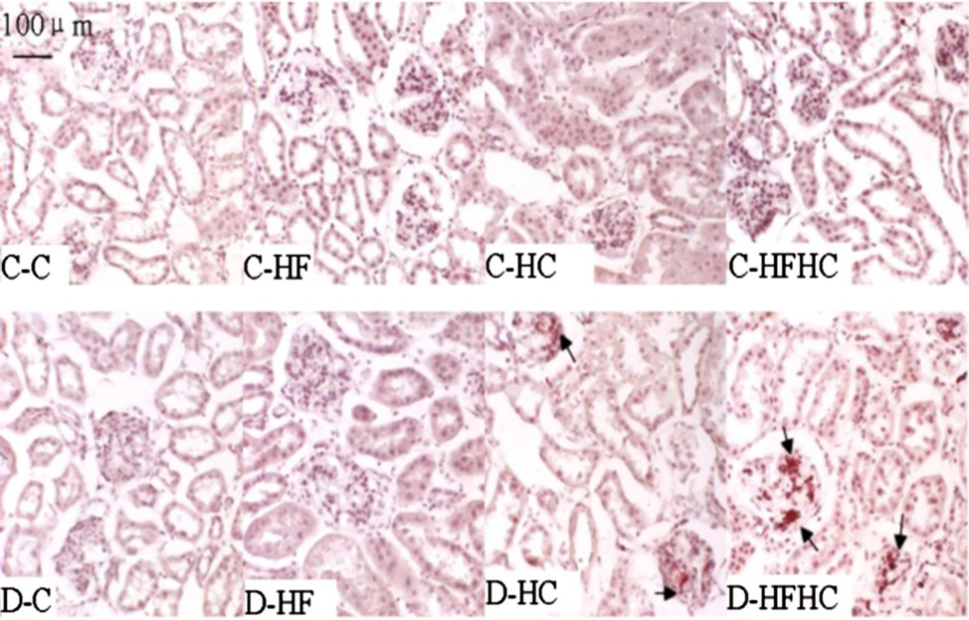
Oil Red O staining from hamsters in each group 4 weeks after a high fat and /or high cholesterol diet(magnification × 200).

**Figure 5:**
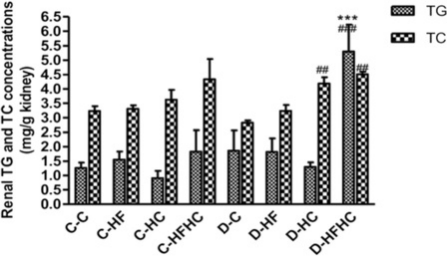
Renal triglyceride and cholesterol concentrations of each group from hamsters in each group.

Renal Oil Red O staining was performed to determine neutral lipid accumulation. The D-HFHC group and D-HC group showed an increased Oil Red O accumulation in glomeruli (Figure 4). There was a significant increase of TG and TC content in kidneys from the D-HFHC group (Figure 5).

### Histopathological examination of kidneys

**Figure 6:**
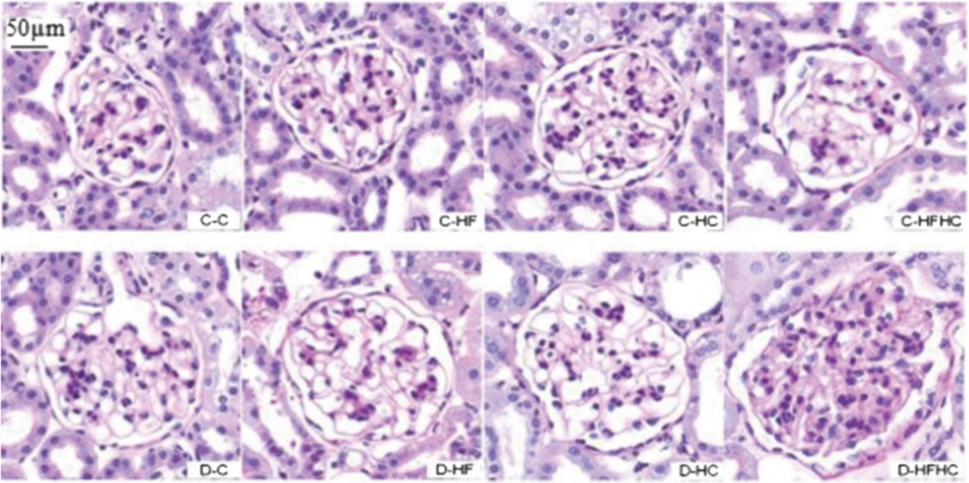
PAS staining of kidney cortex from hamsters in each group 4 weeks after a high fat and /or high cholesterol diet(magnification × 400).

**Figure 7:**
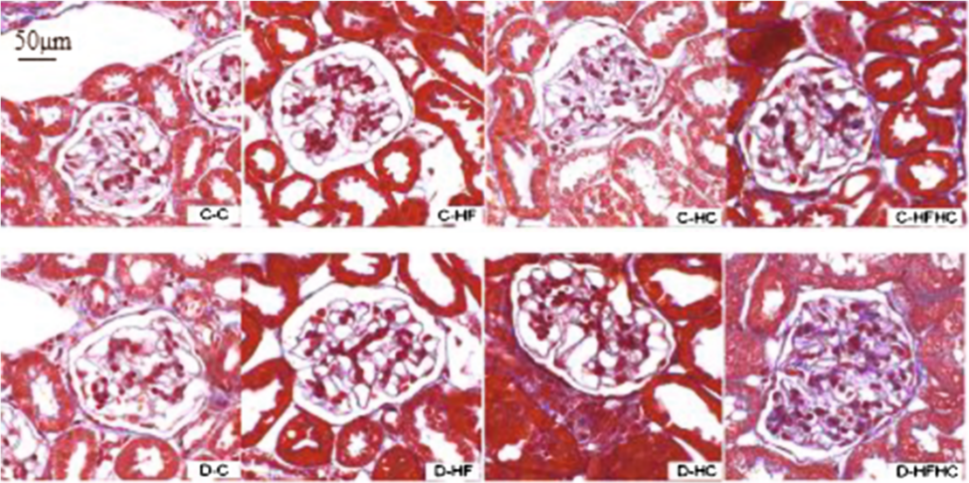
Masson’s trichrome staining of kidney cortex from hamsters in each group 4 weeks after a high fat and /or high cholesterol diet(magnification × 400).

**Figure 8:**
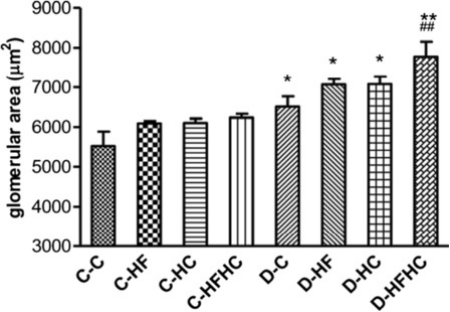
Glomerular areas of hamsters in each groups. n = 6 in each group. ^***^P<0.001, ^**^P< 0.01, ^*^P< 0.05, diabetic vs non-diabetic counterparts; ^###^ P< 0.001, ^##^ P< 0.01, ^#^ P< 0.05 for diabetic with different diets vs D-C group.

Representative kidney sections stained with PAS (Figure 6) and Masson’s trichrome (Figure 7) are shown. No major glomerular pathology changes were observed in any non-diabetic group. In contrast, diabetic hamsters showed significant vacuolar degeneration of glomeruli. With PAS staining, it was observed that diabetic hamsters manifested a slight increase in glomerular surface area compared with corresponding controls. There were significant increases of glomerular surface area (Figure 8), thickness of basement membrane and mesangial expansion in the D-HFHC group (Figure 6) compared with the D-C group. An appreciable accumulation of collagen in glomeruli was also seen in D-HFHC hamsters (Figure 7). The D-HF and D-HC groups did not exhibit mesangial expansion and glomerular collagen deposition (Figures 6, and 7).

### Renal gene expression

**Figure 9:**
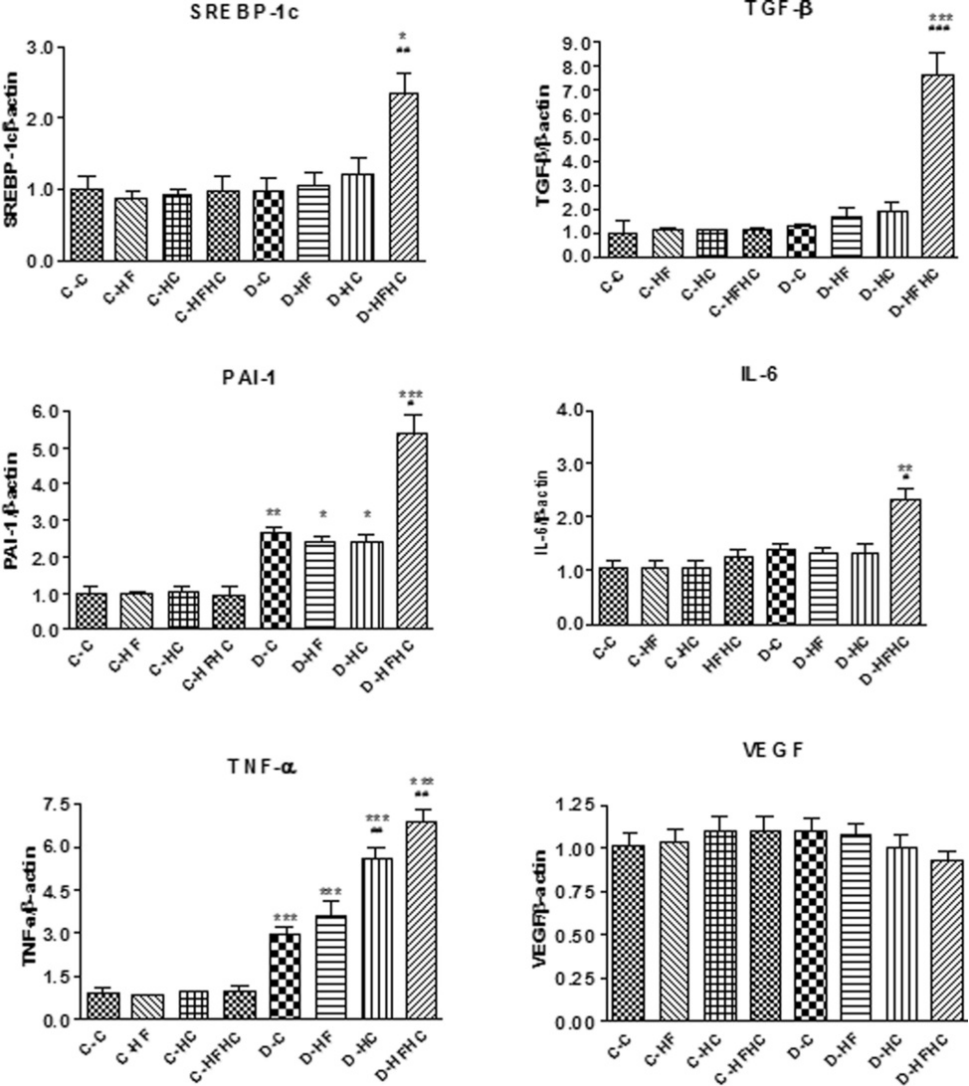
**Effects of STZ-induced diabetes on glomerular gene expression.** ^***^ P<0.001, ^**^P< 0.01, ^*^P< 0.05 diabetic vs non-diabetic counterparts; ^###^ P<0.001, ^##^ P<0.01, ^#^ P<0.05, diabetic with different diets vs D-C group.

In diabetic groups, renal plasminogen activator inhibitor (PAI-1) and tumor necrosis factor-alpha (TNF-α) mRNA expression were significantly up-regulated compared with non-diabetic groups. Expression of these genes increased significantly in the D-HFHC group compared with other diabetic groups (Figure 9). Gene expression of sterol regulatory element binding protein-1c (SREBP-1c) had a fold increase in the D-HFHC group compared with the D-C group (Figure 9). D-HFHC hamsters showed an increase in abundance of renal mRNA of transforming growth factor-beta (TGF-β) and interleukin 6 (IL-6). Diabetic hamsters fed other diets did not show any change (Figure 9). Renal vascular endothelial growth factor (VEGF) mRNA expression did not change in any group (Figure 9).

## Discussion

To the best of the authors’ knowledge, this is the first study to report an animal model with severe hypertriglyceridemia and hypercholesterolemia in non-transgenic and non-surgical T1DM animals. The model was achieved in hamsters induced with short-term high-fat/high cholesterol diets and low dose STZ-treated. The results suggested that T1DM model hamsters developed severe hypertriglyceridemia, hypercholesterolemia, proteinuria with mesangial matrix accumulation and produced accelerated renal injury in 4 weeks. Postprandial plasma TG levels increased 110 mmol/L in the HFHC group , which was five times the previous value reported in which hamsters were fed a saturated fat, non purified diet (10 g butter/100 g diet, 0.1 g cholesterol/100 g) [26]. One rat model was previously established after 15 weeks that showed microalbuminuria, an increased creatinine clearance rate and mesangial expansion using a combination of a high-fat diet, low dose STZ treatment and heminephrectomy [27]. Another hamster model was induced to moderate diabetes and severe glomerulosclerosis with a saturated-fat diet in an experiment lasting 20 weeks [28]. Comparing to these previous reports, the current study suggested that this diabetic hamsters model was a more cost effective, easier to develop and most suited for pathophysiology studies on renal injuries at the early stage of T1DM.

Both insulinotropic (glipizide) and insulin-sensitizing (pioglitazone) agents failed to reduce plasma Glu, TG, TC and insulin levels, when compared with vehicle-treated diabetic hamsters. This indicated that hamsters fed high-fat/high cholesterol diets, and a low dose of STZ (30 mg/kg) resembled T1DM. Previous studies have mainly focused on the effects of dyslipidemia in the development and progression of renal disease in T2DM [27,29-31]. However, clinical studies demonstrated that dyslipidemia is mandatory in patients with T1DM [32,33]. Thus, our initial attempts were directed towards finding the threshold dose of STZ that was low enough to guarantee the development of T1DM in high-fat/high cholesterol hamsters with circulating insulin deficiency. In this study, low dose of STZ (30 mg/kg) succeeded in producing hyperglycemia in T1DM hamsters due to the direct pancreatic beta cell destruction and the resulting insulin deficiency, while T2DM rats induced by low dose of STZ (40 mg/kg) was primarily the consequence of insulin resistance [27]. These distinct differences between hamsters and rats meant that the hamsters might well be mimicking biochemical and physical characteristics of people with diabetes.

In the current study, it was observed that the D-HFHC group developed severe hypertriglyceridemia and hypercholesterolemia with significantly increasing in plasma TC and TG, which was consistent with previous reports [18,34]. The severe hypertriglyceridemia observed in the hamsters may be due to increased absorption and formation of triglycerides in the form of chylomicrons following exogenous consumption of a diet rich in fat, or through increased endogenous production of TG-enriched hepatic very low density lipoprotein and decreased TG uptake in peripheral tissues [35]. Hypercholesterolemia may be attributed to increased dietary cholesterol absorption from the small intestine following the intake of fat in a diabetic condition [36]. One striking finding of the current experiments was the synergistic effects of dietary fat and cholesterol on plasma TG and TC levels in T1DM developing severe hypertriglyceridema and hypercholesterolemia. The results indicated that plasma TC could be notably elevated by dietary cholesterol combined with saturated fat and total fat in the diet. A previous study reported that dietary hypercholesterolemia had little effect on plasma TC in rats [37]. The current study suggested that combined dietary fat and cholesterol notably improved plasma TC levels in T1DM hamsters more than either fat or cholesterol alone, which was in agreement with STZ-induced (insulin-deficient) diabetes in rats enhancing fat and cholesterol absorption and reducing cholesterol synthesis [38]. This animals model also showed insulin deficiency, as determined by anti-diabetic compound effects, which was different to a previously reported saturated-fat diet hamster model with insulin resistance [28]. The current study suggested that low doses of STZ could induce T1DM in hamsters by recruiting immune cells for immune-mediated apoptotic cell death. Hence, this model with the involvement of both insulin deficiency and obvious body weight loss in the development of diabetes could be suitable for studying the pathophysiology of T1DM.

Logan et al. examined the effects of a high-fat diet on renal function in STZ-treated rats and observed that there was no difference in urinary protein excretion between diabetic and control rats [39]. Cooper et al. added cholesterol to the diet of both STZ-injected (55 mg/kg) and normal rats and observed that 32 weeks on a high cholesterol diet did not change urinary albumin levels in normal or diabetic rats [40]. Being inconsistent with these findings, the results of this study showed that urinary protein excretion increased significantly in hamsters fed a high-fat/high-cholesterol diet. The detrimental effects of a high-fat diet on renal function might be triggered by a high-cholesterol diet. Creatinine clearance has been used for many decades to estimate glomerular filtration rate, and is often used for the initial evaluation of renal diseases, such as glomerulonephritis [41]. In the current study, creatinine clearance was significantly elevated in high fat/high cholesterol diabetic hamsters induced by STZ which was also an indication of renal dysfunction. Given that renal lipid deposition and renal histopathological lesions were observed in high-fat/high-cholesterol diabetic hamsters, a high-fat/high-cholesterol diet was considerably effective at inducing structural and functional abnormalities in the diabetic kidney. The results also suggested that hamsters fed a high fat diet for a short period of time fail to produce an evident renal injury, while diabetic hamsters fed with high-fat/high-cholesterol diet exhibited obviously histopathological changes even after 4 weeks of dietary manipulation. Glomerulosclerosis was also seen in this group in long term treatment (up to 2 months). Therefore, the synergistic effects of hypertriglyceridemia and hypercholesterolemia led to produce accelerated renal injury in T1DM hamsters in a short period of time.

Though it is known that dysregulation of renal lipid metabolism plays an important role in the pathogenesis of diabetic nephropathy, the underlying lipids mechanisms mediating glomerulosclerosis have not been fully elucidated. One possible explanation is that lipid can stimulate mesangial cells to proliferate, produce excess basement membrane material and result in the development of sclerotic glomerular lesions [42]. Lipid extraction analysis showed lipid deposition in the kidney of the D-HFHC group, and was further confirmed by Oil Red O staining. This result could possibly be explained by up-regulation in renal gene expression of SREBP-1c. SREBP-1c is known to be a key transcription factor for the regulation of lipogenic gene expression and increase of triglyceride storage in the liver [43,44]. In the current study, renal expression of SREBP-1c mRNA showed a one fold increase in hamsters in the D-HFHC group, which possibly resulted in lipid accumulation in the kidney by TGF-β inhibiting glomerular endothelial, epithelial, and mesangial proliferation and mediating the hypertrophic and fibrotic/sclerotic manifestations of diabetic nephropathy. Our results also showed that increased expression of TGF-β, PAI-1, and pro-inflammatory cytokines(TNF-α and IL-6) were found in the D-HFHC group, which was in alignment with a previous report [43]. Proteinuria is considered the earliest clinical indicator of incipient diabetic nephropathy, and podocyte-derived VEGF is involved in the proteinuria of diabetes [45]. Increased expression of VEGF in the glomerular, and tubulointerstitium were reported in STZ induced mice and rat models of diabetic nephropathy, as well as experimental models of T2DM [46,47]. However, in the current study, renal VEGF expression in hamsters in the D-HFHC group did not increase, and even showed a tendency to decrease, which was similar to human diabetic nephropathy. Lindenmeyer et al. found a significant decrease in VEGF expression on mRNA and protein levels in human diabetic nephropathy, and suggested that a lack of VEGF, rather than an excess of VEGF, might contribute to the progressive disease in human diabetic nephropathy [48]. The results of the current study indicateed that a lack of VEGF in this model might also induce renal injury in type 1 diabetic hamsters.

## Conclusions

In conclusion, the results indicated that severe hypertriglyceridemia and hypercholesterolemia produced accelerated renal injury in hamsters during the early development of T1DM induced by short-term high-fat/high-cholesterol diet and low dose STZ treatment. TG and TC deposition, glomerulosclerosis, mesangial hyperplasia, and proteinuria might combine to cause renal injury in T1DM hamsters in a short period of time. The underlying molecular mechanism might be increased expression of SREBP-1c associated with TGF-β, TNF-α, IL-6 and PAI-1. Furthermore, this study provides a diabetic animal model that is more cost effective, easier to develop and most suited for researching the pathogenesis and treatment of diabetes and associated dyslipidemia.

## Additional file

Additional file 1:
**The ARRIVE guidlines checklist.**

## Abbreviations

Glu: Plasma glucose
IL-6: Interleukin-6
PAS: Periodic acid-Schiff
PAI-1: Plasminogen activator inhibitor-1
T1DM: Type 1 diabetes mellitus
T2DM: Type 2 diabetes mellitus
TC: Total cholesterol
TG: Triglyceride
TGF-β: Transforming growth factor-beta
TNF-α: Tumor necrosis factor-alpha
SREBP-1c: Sterol regulatory element binding protein-1c
STZ: Streptozotocin
VEGF: Vascular endothelial growth factor
