# Supplementary material for: Severe hypertriglyceridemia and hypercholesterolemia accelerating renal injury: a novel model of type 1 diabetic hamsters induced by short-term high-fat / high-cholesterol diet and low-dose streptozotocin
Source: BMC Nephrol. 2015 Apr 11;16:51. doi: 10.1186/s12882-015-0041-5 (PMC4429331; doi:10.1186/s12882-015-0041-5)
Supplement: Additional file 1: — The ARRIVE guidlines checklist. [file 12882_2015_41_MOESM1_ESM.pdf]

The ARRIVE Guidelines

Animal Research: Reporting *In Vivo* Experiments

	Item	Recommendation	Section/Paragrah
**TITLE**	**1**	Provide as accurate and concise a description of the content of the article as possible.	Severe hypertriglyceridemia and hypercholesterolemia accelerating renal injury: A novel model of type 1 diabetic hamsters induced by short-term high-fat / high-cholesterol diet and low-dose streptozotocin.
**ABSTRACT**	**2**	Provide an accurate summary of the background, research objectives, including details of the species or strain of animal used, key methods, principal findings and conclusions of the study	BACKGROUND AND PURPOSE: Hyperlipidemia is thought to be a major risk factor for the progression of renal diseases in diabetes. Recent studies have shown that lipid profiles are commonly abnormal early on type 2 diabetes mellitus (T2DM) with diabetic nephropathy. However, the early effects of triglyceride and cholesterol abnormalities on renal injury in type 1 diabetes mellitus (T1DM) are not fully understood and require reliable animal models for exploration of the underlying mechanisms. Our aim was to establish a novel, non-transgenic, and non-surgical animal model induced by a short-term high-fat/high-cholesterol diet and low-dose streptozotocin(STZ) treatment in Golden Syrian hamsters, and to explore the mechanisms underlying severe hypertriglyceridemia and hypercholesterolemia producing accelerated renal injury in this model.<br><br>EPERIMENTAL APPROACH: Male Golden Syrian hamsters (100–110 g) were rendered diabetes by intraperitoneal injections of streptozotocin (STZ) on consecutive 3 days at dose of 30 mg/kg, Ten days after STZ injections, hamsters with a plasma Glu concentration more than 12mmol/L were selected as insulin deficient ones and divided into four groups (D-C, D-HF, D-HC, and D-HFHC), and fed with commercially available standard rodent chow, high-fat diet, high-cholesterol diet, high-fat and cholesterol diet respectively, for a period of four weeks. Finally, we measured plasma biochemical parameters (Glu, TC, TG,Insulin and creationine) and urinary protein excretion, analysed renal histologicology, and renal gene expression (TNF-α, SREBP-1c, PAI-1, IL-6, TGF-β,VEGF, β-actin,18S) by real-time PCR.

			KEY RESULTS: We have demonstrated that severe hypertriglyceridemia and hypercholesterolemia produced accelerated renal injury in hamsters during the early development of T1DM induced by short-term high-fat/high-cholesterol diet and low dose STZ treatment.<br><br>CONCLUSIONS AND IMPLICATIONS: These results highlighted a diabetic animal model that was more cost effective, easier to develop and most suited for researching the pathogenesis and treatment of diabetes and associated dyslipidemia.
**INTRODUCTION**			
Background	**3**	a. Include sufficient scientific background (including relevant references to previous work) to understand the motivation and context for the study, and explain the experimental approach and rationale.<br><br>b. Explain how and why the animal species and model being used can address the scientific objectives and, where appropriate, the study's relevance to human biology.	Hyperlipidemia is thought to be a major risk factor for the progression of renal diseases in diabetes. Recent studies have shown that lipid profiles are commonly abnormal early on type 2 diabetes mellitus (T2DM) with diabetic nephropathy. However, the early effects of triglyceride and cholesterol abnormalities on renal injury in type 1 diabetes mellitus (T1DM) are not fully understood and require reliable animal models for exploration of the underlying mechanisms.<br><br>For this purpose, we selected hamster models as an important tool for studying lipid metabolism because of their similarity to humans in terms of lipid utilization and high susceptibility to dietary cholesterol and fat.
Objectives	**4**	Clearly describe the primary and any secondary objectives of the study, or specific hypotheses being tested.	Therefore ,the objective of this study were to establish a novel, non-transgenic, and non-surgical animal model induced by a short-term high-fat/high-cholesterol diet and low-dose streptozotocin(STZ) treatment in Golden Syrian hamsters, and to explore the mechanisms underlying severe hypertriglyceridemia and hypercholesterolemia producing accelerated renal injury in this model.
METHODS			
Ethical statement	**5**	Indicate the nature of the ethical review permissions, relevant licences (e.g. Animal [Scientific Procedures] Act 1986), and national or institutional guidelines for the care and use of animals, that cover the research.	All animal procedures were approved by the Institutional Committee on the Use of Live Animals in Teaching and Research at Zhejiang Academy of Agricultural Sciences. 'Principles of Laboratory Animal Care' (NIH Publication No.85-23, revised 1996) were followed.

Study design	**6**	For each experiment, give brief details of the study design including:<br><br>a. The number of experimental and control groups.<br><br>b. Any steps taken to minimise the effects of subjective bias when allocating animals to treatment (e.g. randomisation procedure) and when assessing results (e.g. if done, describe who was blinded and when).<br><br>c. The experimental unit (e.g. a single animal, group or cage of animals). A time-line diagram or flow chart can be useful to illustrate how complex study designs were carried out.	In experiment for determination of threshold dose of STZ on hamsters producing insulin deficient, four groups of 24 animals were studied by intraperitoneal injections of STZ on 3 consecutive days, at dose of either 20 mg/kg, 30 mg/kg, 40 mg/kg or 50 mg/kg once daily in 0.05 M citrate buffer (pH 4.5). The control group (n=6) was given 0.05 M citrate buffer (pH 4.5) without STZ. In experiment for establishing the model of hamsters induced by STZ, hamsters with a plasma Glu concentration more than 12mmol/L were selected as insulin deficient ones for subsequent experiments. Twenty-four STZ-treated hamsters were randomly divided into four groups (n= 6 in each group) according to the type of diet they recceived: either standard rodent chow (D-C), high-fat diet (D-HF), high-cholesterol diet (D-HC) or high-fat and high-cholesterol diet (D-HFHC). Twenty-four control hamsters were also divided into four groups (n=6 in each group) according to the different diets they received(C-C, C-HF, C-HC or C-HFHC). In experiment for determination of high-fat/high-cholesterol feeding and STZ-treated type1 diabetic hamsters through anti-diabetic compound effects, high-fat/high-cholesterol and STZ-treated hamsters (n=18) were randomly divided in three groups: A. treated with pioglitazone; B. a single dose of glipizide; C.the control group was orally given vehicle 1% Na-CMC.<br><br>In all experiments, animals were randomized into treatment groups by picking numbers out of a hat. In the study, n refers to number of animals (six animals each group).
Experimental procedures	**7**	For each experiment and each experimental group, including controls, provide precise details of all procedures carried out. For example:<br><br>a. How (e.g. drug formulation and dose, site and route of administration, anaesthesia and analgesia used [including monitoring], surgical procedure, method of euthanasia). Provide details of any specialist equipment	All hamsters were rendered diabetes by intraperitoneal injections of STZ. Prior to exsanguinate by cutting the abdominal aorta, animals were given an i.p.injection of 900μL/100g 1% pentobarbital sodium as anesthetic.<br><br>All experiments were conducted in the light phase.<br><br>The animals were tested in rectangle shaped cages (370 mm long, 215 mm wide and 170 mm high).<br><br>The intraperitoneal (i.p.) route may be used for agents to prolong during of action.

		used, including supplier(s).<br>b. When (e.g. time of day).<br>c. Where (e.g. home cage, laboratory, water maze).<br>d. Why (e.g. rationale for choice of specific anaesthetic, route of administration, drug dose used).	
Experimental animals	**8**	a. Provide details of the animals used, including species, strain, sex, developmental stage (e.g. mean or median age plus age range) and weight (e.g. mean or median weight plus weight range).<br>b. Provide further relevant information such as the source of animals, international strain nomenclature, genetic modification status (e.g. knock-out or transgenic), genotype, health/immune status, drug or test naïve, previous procedures, etc.	Male Golden Syrian hamsters (100–110 g), aged 2 months old, were included (n=94) All animals were purchased from Beijing Vital River Laboratory Animal Technology Co.,Ltd and acclimatized for at least 48 h. Vendor health reports indicated that the animals were free of known viral, bacterial and parasitic pathogens.
Housing and husbandry	**9**	Provide details of:<br>a. Housing (type of facility e.g. specific pathogen free [SPF]; type of cage or housing; bedding material; number of cage companions; tank shape and material etc. for fish).<br>b. Husbandry conditions (e.g. breeding programme, light/dark cycle, temperature, quality of water etc for fish, type of food, access to food and water, environmental	Hamsters were housed with an inverse 12 hours day-night cycle with lights on at 8:00pm in a temperature (23±1℃) and humidity (45±5%) controlled room.<br>All hamsters were allowed free access to water and a maintenance diet containing 4% fat, 24% protein and 4.5% crude fiber in a 12-hour light/dark cycle, with room temperature at 23±1℃.<br>All cages contained wood shavings and bedding for environmental enrichment.

		enrichment).<br>c.Welfare-related assessments and interventions that were carried out prior to, during, or after the experiment.	
Sample size	**10**	a. Specify the total number of animals used in each experiment, and the number of animals in each experimental group.<br>b. Explain how the number of animals was arrived at. Provide details of any sample size calculation used.<br>c. Indicate the number of independent replications of each experiment, if relevant.	In experiment for determination of threshold dose of STZ on hamsters producing insulin deficient, thirty animals were divided in five group of six each. Animals of group Ⅰ, Ⅱ, Ⅲ, Ⅳ were treated with STZ at dose of 20 mg/kg, 30 mg/kg, 40 mg/kg or 50 mg/kg once daily in 0.05 M citrate buffer (pH 4.5). The control group was given 0.05 M citrate buffer (pH 4.5) without STZ. In experiment for establishing the model of hamsters induced by STZ, twenty-four STZ-treated hamsters were divided into four groups of six each. Animals of group D-C, D-HF, D-HC, or D-HFHC were fed with standard rodent chow (D-C), high-fat diet (D-HF), high-cholesterol diet (D-HC) or high-fat and high-cholesterol diet (D-HFHC), respectively. Twenty-four control hamsters were also divided into four groups of six each and feed with different diets they received(C-C, C-HF, C-HC or C-HFHC). In experiment for determination of high-fat/high-cholesterol feeding and STZ-treated type1 diabetic hamsters through anti-diabetic compound effects, eighth STZ-treated animals were divided in three group of six each. Animals of group A and B were treated with pioglitazone and glipizide, respectively. The control group was orally given vehicle 1% Na-CMC.<br>Sample size calculations were performed in G Power (Faul, Erdfelder, Lang and Buchner, 2007).Mean triglyceride level of healthy hamsters weighing 100 to 110 g. was 102.9 mg/dl with a standard deviation of 16.2 mg/dl (the noise). A difference in triglyceride of 30 mg/dl (the signal) or more would be of clinical importance. Then the signal/noise ratio would be 30/16.2 = 1.8. To achieve power = 80% and alpha = 0.05 to detect this difference would require 6 animals/group and a total of 96 animals.<br>The experiment was repeated, and data were pooled.
Allocating animals to	**11**	a. Give full details of how animals were allocated to experimental groups, including	For experiments using animals, hamsters were randomly allocated to treatment groups in order i.e. hamster 1 group Ⅰ, hamster 2 group Ⅱ, hamster 3 group Ⅲ,

experimental groups		randomisation or matching if done.<br><br>b. Describe the order in which the animals in the different experimental groups were treated and assessed.	hamster 4 group Ⅰ, etc., thus ensuring the median of each group was similar prior to testing.<br><br>For all experiments, sequences of A–B–C-D-E-F then F-E-D-C–B–A (letters assigned to mask the cage labels during testing) were used to select animals.
Experimental outcomes	**12**	Clearly define the primary and secondary experimental outcomes assessed (e.g. cell death, molecular markers, behavioural changes).	Two primary outcome were establishment of a diabetic animal model and severe hypertriglyceridemia and hypercholesterolemia produced accelerating renal injury. Secondary outcome were found that the underlying molecular mechanism might be increased expression of SREBP-1c associated with TGF-β, TNF-α, IL-6 and PAI-1.
Statistical methods	**13**	a. Provide details of the statistical methods used for each analysis.<br><br>b. Specify the unit of analysis for each dataset (e.g. single animal, group of animals, single neuron). c. Describe any methods used to assess whether the data met the assumptions of the statistical approach.	Statistical analysis using 2-way ANOVA followed by a Fisher test was performed for between groups (i.e., body weight, plasma glucose, total cholesterol, triglyceride, glomerular areas).<br><br>For each test, the experimental unit was an individual animal.
RESULTS			
Baseline data	**14**	For each experimental group, report relevant characteristics and health status of animals (e.g. weight, microbiological status, and drug or test naïve) prior to treatment or testing. (This information can often be tabulated).	The animals' health status was monitored throughout the experiments by a health surveillance programme according to Chinese Laboratory Animal Management guidelines. The hamsters were free of all viral, bacterial, and parasitic pathogens listed in the Chinese Laboratior animal- Microbiological standards and monitoring (GB 14922.2-2011)
Numbers analyzed	**15**	a. Report the number of animals in each group included in each analysis. Report absolute numbers (e.g. 10/20, not 50%2).<br><br>b. If any animals or data were not included in the analysis, explain why	All Six animals in each group were included in each analysis.

Outcomes and estimation	**16**	Report the results for each analysis carried out, with a measure of precision (e.g. standard error or confidence interval).	In accordance with the ARRIVE guidelines, we have reported measures of precision, confidence, and n to provide an indication of significance. Cages were randomly assigned to each hamster.
Adverse events	**17**	a. Give details of all important adverse events in each experimental group<br>b. Describe any modifications to the experimental protocols made to reduce adverse events.	In our study, β-actin levels are often affected by diabetes. The target mRNA levels was normalized β-actin and 18S together (geometric mean) as reference genes.<br>We assessed the values of expression stability of B actin and 18S and coefficient of variation of their normalized relative quantities respectively.
DISCUSSION			
Interpretation/scientific implications	**18**	a. Interpret the results, taking into account the study objectives and hypotheses, current theory and other relevant studies in the literature. c. Describe any implications of your experimental methods or findings for the replacement, refinement or reduction (the 3Rs) of the use of animals in research.	The aim of our study was to establish a stable model of renal injury with the aspects of early T1DM kidney disease These aspects were severe hypertriglyceridemia, hypercholesterolemia, proteinuria with mesangial matrix accumulation, upgraded creatinine clearance, significant cholesterol and triglyceride deposition, and increasing glomerular surface area, thickness of basement membrane and mesangial expansion. The mRNA levels of sterol regulatory element binding protein-1c, transforming growth factors-β, plasminogen activator inhibitor-1, tumor necrosis factor-α and interleukin-6 in the D-HFHC group were significantly up-regulated compared with control groups.<br>A limitation of this study is the fact that only male hamsters were used, since sex could potentially have affected these aspects of ypertriglyceridemia, hypercholesterolemia, proteinuria with mesangial matrix accumulation, creatinine clearance, cholesterol and triglyceride deposition, and glomerular surface area, thickness of basement membrane and mesangial expansion.<br>The new finding shows potential for considerably reducing the number of animals, because the established techniques for induction of renal injury by surgical in animals have lead to a substantial mortality rate.

Generalisability/translation	**19**	Comment on whether, and how, the findings of this study are likely to translate to other species or systems, including any relevance to human biology	The findings of this study are likely to be similarities of severe hypertriglyceridemia and hypercholesterolemia accelerating renal injury in human type 1 diabetes mellitus.
Funding	**20**	List all funding sources (including grant number) and the role of the funder(s) in the study.	This work was funded by the Zhejiang Important National Science and Technology Specific Projects (2011C12024), Veterinary Medicine Discipline Program of Southwest University for Nationalities (2011XWD-S0906) and Zhejiang Academy of Agricultural Sciences scientific and technological innovation promotion project.
